# Editorial: Community series in mental illness, culture, and society: dealing with the COVID-19 pandemic, volume VIII

**DOI:** 10.3389/fpsyt.2024.1434405

**Published:** 2024-06-11

**Authors:** Renato de Filippis, Samer El Hayek, Mohammadreza Shalbafan

**Affiliations:** ^1^ Psychiatry Unit, Department of Health Sciences, University Magna Graecia of Catanzaro, Catanzaro, Italy; ^2^ Medical Department, Erada Center for Treatment and Rehabilitation in Dubai, Dubai, United Arab Emirates; ^3^ Mental Health Research Center, Psychosocial Health Research Institute, Department of Psychiatry, School of Medicine, Iran University of Medical Sciences, Tehran, Iran

**Keywords:** coronavirus, healthcare professionals, mental health care, mental disorders, psychiatry, psychological distress, public health, SARS-CoV-2

Five years after the coronavirus spread, much has been written and discussed about the impact that its clinical manifestation, viz. the Coronavirus Disease 2019 (COVID-19), has had on all areas of life, research, and medicine (1–3). However, since it was the collective event of greatest global and contemporary relevance in the last century, what is most relevant after overcoming the acute and sub-acute medical emergencies is its cultural and social consequences, and how different countries, minorities, special, and fragile populations have faced and adapted to this phenomenon (4–6). Indeed, COVID-19 has represented a watershed moment for many generations, testing often unprepared health systems and widening personal and systemic suffering, but also accelerating processes of technological innovation and connectivity and intensifying the sense of global community in public health (7–9).

This eighth and final volume of our extensive Research topic collection *Mental illness, culture, and society: dealing with the COVID-19 Pandemic* (10–16) closes this widespread literature compendium, which overall comprises 167 articles addressing what COVID-19 has meant for global public health, from a cultural and social viewpoints (17).

Twelve articles assessed the impact of COVID-19 on healthcare professionals. Among them, Xu et al. evaluated the mental health status of community frontline medical workers after the normalized management of COVID-19 in Sichuan, China. Along the same lines, the study conducted by Schaffler et al. tried to explore burdens, resources, and determinants of good or poor well-being among Austrian psychotherapists. Büssing and Baumann focused on the experience of loss and grief among people from Germany who lost their relatives during the pandemic, with a reflection on the impact of the support provided by healthcare professionals. Through a longitudinal study design, Loureiro et al. also studied the Brazilian healthcare workers’ emotional burden and the effects on professional fulfillment at the end of the third wave of COVID-19. Fatima et al. conducted an in-depth analysis of the moral injury among healthcare providers in Pakistan during the pandemic. Lam et al. launched a national survey in China to study the prevalence of COVID-19 fear and its association with quality of life and network structure among Chinese mental health professionals after ending China’s dynamic zero-COVID policy. On the other hand, in another survey study in China, the group led by Feng et al. explored the workload change and depression among emergency medical staff after the open policy during the pandemic. A qualitative cross-sectional analysis by Baranowski et al. evaluated COVID-19 imagery in scientific literature and its use for people working in the German healthcare sector. In another survey, Vaillant-Ciszewicz et al. studied the psychological impact of the first lockdown on French nursing homes, particularly the impact on psychologists, psychomotors therapists, and occupational therapists. Wang et al. managed a large-scale cross-sectional study about the prevalence of anxiety and associated factors among frontline nurses following the pandemic in China. Still, the work by Sanchez-Plazas et al. highlights the psychological impact of the COVID-19 pandemic on physicians in Puerto Rico. Finally, the last paper in this specific sub-topic emanates from Nowicki et al., who focused on the relationship between the strength of religious faith and spirituality with post-traumatic growth among nurses caring for patients with COVID-19 in eastern Poland.

Many articles discussed the impact of COVID-19 on students, trainees, and residents. In this regard, the study by Ashiq et al. reported on the levels of depression, anxiety, stress, and fear of COVID-19 among Bangladeshi medical students during the first wave of the pandemic in the country. The group led by Anteneh et al. in Ethiopia focused on the psychological impact of the pandemic and associated factors among college and university students in 2022. The paper by Serrano et al. explored sociodemographic characteristics, social support, and family history as risk factors for depression, anxiety, and stress among young adult senior high school students in the Philippines. Alternatively, Wei et al. evaluated the current status of e-learning, personality traits, and coping styles among medical students during the pandemic. Cebrino and Portero de la Cruz wrote a piece about the psychological impact of COVID-19 and its determinants among Spanish university students. Zhang et al. wanted to better understand which psychological status and related factors of resident physicians were more relevant during the release of COVID-19 pandemic restrictions in China. In the article by Rahgozar and Giménez-Llort, findings highlight the design and effectiveness of an online group logotherapy intervention on the mental health of Iranian international students residing in European countries during the pandemic. Ding et al. published a paper about the prospective associations between time management tendency, negative emotions, and problematic smartphone use among Chinese nursing students. Still, Wagner et al. studied mental distress, food insecurity, and university student dropout during the COVID-19 pandemic in South Africa. Finally, the last paper in this subtopic emerges from Fu et al. who validated a scale evaluating multicultural personality traits of Chinese university students and their effects on psychological adjustment in the aftermath of COVID-19 in Shanghai.

The richest group of articles is that of 15 manuscripts that discuss special, fragile, or minority populations during the pandemic. Usher et al. explored the relationship between mental health and the use of medicare benefits follow-up mental health services by Indigenous people in Australia during COVID-19. Furthermore, the team led by Busili et al. studied COVID-19 exposure and depression-anxiety levels among Saudi adults in the Jazan region, in a sample size predominantly made up of female and undergraduate individuals. Steinhausen-Wachowsky et al. evaluated the stability of psychological well-being during the pandemic among people with an anthroposophical worldview, with a specific reflection on the influence of wondering awe and perception of nature as resources. Nadeem et al. shared findings about the impact of empathy, sensation seeking, anxiety, uncertainty, and mindfulness on intercultural communication in China during COVID-19. It is also interesting to read about the impact of resilience on the mental health of military personnel during the pandemic in the paper by Cao et al. The retrospective cross-sectional study conducted by Adam et al. found an increase of new-onset psychiatric disorders in a psychiatric emergency department in Berlin, Germany during the second wave of the pandemic. Lanchimba et al. explored potential factors influencing domestic violence during COVID-19 restrictions. Ibrahim et al. conducted a cross-sectional study about the relationship between depression and death anxiety among patients undergoing hemodialysis during the pandemic in Palestine. Another relevant paper by Gu et al., conducted in South Korea, reported on which factors can influence the coping skills of middle-aged adults during COVID-19. Again from South Korea, Kim et al. examined the relationship between sleep quality and depressive symptoms in women engaged in soccer. A four-year prospective study run in Taiwan by Huang et al. evaluated the predictive effects of prepandemic sexual stigma, affective symptoms, and family support on the fear from COVID-19 among lesbian, gay, and bisexual individuals. Bühler and Willmund investigated, during the third wave of the pandemic, the deployment-related quarantining as a risk or resilience factor for German military service members. The study led by Liu et al. estimated the prevalence of COVID-19 fear and its association with quality of life among fire service recruits after ceasing the dynamic zero-COVID policy. Dones and Ciobanu studied the older adults’ experiences of wellbeing during the pandemic in a comparative qualitative study design, with a peculiar focus on coping mechanisms in Italy and Switzerland. Lastly, Wei et al. estimated the prevalence of depressive symptoms and its correlates among individuals in China who self-reported SARS-CoV-2 infection after optimizing the COVID-19 response.

Nine studies were conducted in specific locations or areas. Among them, in Japan Saito et al. studied the age group differences in psychological distress and leisure-time exercise/socioeconomic status during the pandemic. Krawczyk-Suszek and Kleinrok published a paper on the quality of life of a healthy Polish population in association with specific sociodemographic factors during COVID-19. Moya-Salazar et al. ran a systematic review tackling the mental health situation in rural Andean populations in Latin America during the pandemic. Abdel-Rahman et al. proposed a series of predictors of mental health issues during the outbreak in Egypt, mainly highlighting food security, incomes, and livelihoods. In a Mendelian randomization study, Xue et al. investigated the susceptibility and severity of COVID-19 and the risk of psychiatric disorders in European populations. Chu and Lee conducted a propensity score matching analysis on depressive symptoms among people under COVID-19 quarantine or self-isolation in South Korea. In a study from Saudi Arabia, Al-Johani et al. evaluated post-COVID-19 fatigue and health-related quality of life in the general population. Zenba et al. published a paper in Japan on psychological distress among older adults due to fear from COVID-19, mainly via lifestyle disruption and leisure restriction. Finally, Fernandes et al. studied stress, anxiety, and depression trajectories during the first wave in Portugal, through some drives such as resilient, adaptive, and maladaptive responses.

Four studies about specific treatments or home therapies were included in this volume. In particular, the work by Moreno-Alonso et al. analyzed patients’ satisfaction and outcomes of crisis resolution home treatment for the management of acute psychiatric crises during the COVID-19 pandemic in Madrid. Sandra et al. proposed a peculiar pilot study to apply a two-week home exercise program targeting depressive symptoms in the coronavirus crisis context. On the other hand, Jiang et al. published a paper including a multidimensional comparative analysis of help-seeking messages during different stages of the pandemic in China. Finally, Luo et al. discussed the role of diverse forms of art therapy as potentially effective treatment measures for psychiatric symptoms in patients with COVID-19.

Three papers discussed pharmacoeconomics and politics during the pandemic. He et al. studied the potential link between the relaxation of the COVID-19 control policy and residents’ mental health, in view of the mediating role of family tourism consumption in China. In the article by Sarasjärvi et al., the behavioral patterns Western Australians during and after lockdown were analyzed. Lastly, the team led by Stojkovic et al. presented a brief report on the impact of the pandemic on the prescription trend of long-acting injections of paliperidone and risperidone in Serbia.

Two articles dealt with the theme of suicide. Jeremic et al. assessed whether the trend of suicide by self-immolation among a sample of adolescents may have undergone significant changes due to COVID-19. alternatively, Badrfam et al. analyzed data about suicidal thoughts and burnout and their association among healthcare workers in Iran after the fourth wave of the pandemic.

The paper published by Msetfi et al. analyzed pandemic-collected data and 1) assessed the correlation between feelings of control and depression, 2) explored if varying control measures influenced this correlation, and 3) determined if this relationship was altered based on pandemic indicators.

Finally, we included six papers that presented data, reflections, or proposals for the management of the future or long-term COVID-19 consequences. Nisa et al. hypothesized that the COVID-19 pandemic could represent a pivotal moment in global backing for universal health coverage, as indicated by a heightened agreement regarding the government’s roles as a healthcare provider. Ransing et al. discussed, in a positive and optimistic vision, the substantial international scientific collaboration during and after the pandemic, as demonstrated by the increase in multidisciplinary and international scientific literature. Wagner et al. emphasized the necessity of enhancing communication and accessibility to mental health services in higher education. Recommendations and implications for policy and support services were outlined. Mejia et al. conducted a cross-sectional survey examining the prevalence of risk for post-traumatic stress disorder following COVID-19 across 12 countries in Latin America. Through a systematic review, Martínez-Borba et al. directed forthcoming research on psychological interventions for individuals with COVID-19 and post-COVID syndrome, comorbid with concurrent emotional disorders. Finally, in Columbia, Bautista-Gomez et al. examined the country’s specific psychosocial risk profiles to manage future health emergencies.

As of May 2024, the World Health Organization reported more than 775 million confirmed cases of COVID-19 worldwide, and over 7 million deaths directly related to the coronavirus, as well as incalculable damage to physical health, mental health, and quality of life around the world (18–20). At the same time, through the efforts of frontliners, physicians, and researchers, the articles collected in this eighth and previous seven volumes, demonstrate a beacon of hope towards better understanding and facing the challenge of the COVID-19 pandemic and its impact on mental health, allowing to draw robust conclusions and expectations for the years to come (21–23).

## Author contributions

RF: Writing – original draft. SE: Writing – review & editing. MS: Writing – review & editing.
